# Adjusting cotton planting density under the climatic conditions of Henan Province, China

**DOI:** 10.1371/journal.pone.0222395

**Published:** 2019-09-26

**Authors:** Liyuan Liu, Chuanzong Li, Yingchun Han, Zhanbiao Wang, Lu Feng, Xiaoyu Zhi, Beifang Yang, Yaping Lei, Wenli Du, Yabing Li

**Affiliations:** 1 Institute of Cotton Research of Chinese Academy of Agricultural Sciences, Anyang, Henan, China; 2 State Key Laboratory of Cotton Biology, Anyang, Henan, China; Valahia University of Targoviste, ROMANIA

## Abstract

The growth and development of cotton are closely related to climatic variables such as temperature and solar radiation. Adjusting planting density is one of the most effective measures for maximizing cotton yield under certain climatic conditions. The objectives of this study were (1) to determine the optimum planting density and the corresponding leaf area index (LAI) and yield under the climatic conditions of Henan Province, China, and (2) to learn how climatic conditions influence cotton growth, yield, and yield components. A three-year (2013–2015) field experiment was conducted in Anyang, Henan Province, using cultivar SCRC28 across six planting density treatments: 15,000, 33,000, 51,000, 69,000, 87,000, and 105,000 plants ha^−1^. The data showed that the yield attributes, including seed cotton yield, lint yield, dry matter accumulation, and the LAI, increased as planting density increased. Consequently, the treatment of the maximum density with 105,000 plants ha^-1^ was the highest-yielding over three years, with the LAIs averaged across the three years being 0.37 at the bud stage, 2.36 at the flower and boll-forming stage, and 1.37 at the boll-opening stage. Furthermore, the correlation between the cotton yield attributes and meteorological conditions indicated that light interception (LI) and the diurnal temperature range were the climatic factors that most strongly influenced cotton seed yield. Moreover, the influence of the number of growing degree days (GDD) on cotton was different at different growth stages. These observations will be useful for determining best management practices for cotton production under the climatic conditions of Henan Province, China.

## Introduction

As the world population grows, worldwide demand for cotton is increasing and becoming increasingly urgent [1–2]. China is the largest cotton-producing country in the world. Moreover, Henan Province is one of the major cotton growing provinces of China, with more than 400 thousand ha planted [3]. The climate of Henan Province is semiwet during the cotton growing season from April to October. Temperatures are low in the early stage of the growing season, while in the middle and later stages, temperatures are high. Approximately 4500 growing degree days (GDD) (°C) above 10°C and over 1300 hours of incipient radiation are accumulated over the growing season. Moreover, there are approximately 600 mm of precipitation, which is concentrated in July and early August [4–6].

Determining the optimum planting density is regarded as one of the most effective agronomic practices to promote maximum yield [7–10]. Numerous studies have shown that different climatic conditions and planting densities have a great influence on crop growth and population structure [11–15]. The planting density of cotton in China varies depending upon climatic conditions, particularly solar radiation and temperature. For instance, the planting density of cotton in the Yellow River Basin is approximately 3,000 to 5,000 plants ha^-1^, while the planting density in Xinjiang can reach 10,000 to 20,000 plants ha^-1^, which is mainly due to the low annual precipitation, long duration of insolation, and short frost-free period of Xinjiang Province. Therefore, determining the optimum planting density under different climatic conditions would be helpful for cotton production in China and other countries around the world.

Extensive research has demonstrated that proper planting density is the most critical factor for establishing an optimal canopy structure consisting of a good LAI and porosity, which is an important parameter to describe the light transmission capacity of the canopy [16–19]. Researchers have sought for many years to elucidate how planting density is related to the LAI and cotton production [20–24]. They found that the LAI increases with increasing planting density; however, canopy shading occurs when the LAI is excessively high, resulting in reduced cotton production [25–26]. Studies have also shown that the LAI and yield both increase gradually as the planting density increases [27]. Consequently, the relationship between planting density, the LAI and cotton production is still confusing. Therefore, determining the precise LAI at the optimum planting density is of great significance for improving light use efficiency, which is crucial for yield. Moreover, planting density can also affect light interception (LI) and the light extinction coefficient (k), influenced by the crop structure, e.g., the LAI and the orientation of the leaf, which is an important indicator that reflects a crop's ability to intercept light within the crop canopy [28–33]. The study of Xu in 2017 proposed the “optimum planting density” as the one that produced the highest yield. The corresponding k value and LAI were the “optimum k value” and “optimum LAI”, respectively [27].

We conducted a field experiment to determine the optimum planting density, with test densities ranging from 15,000 to 105,000 plants ha^-1^, and the corresponding LAI and yield under the climatic conditions of Henan Province, China and to learn how these climatic conditions influence cotton growth, yield components and yield.

## Materials and methods

### Experimental design

Field experiments were conducted from 2013 to 2015 at an experimental field of the Institute of Cotton Research of the Chinese Academy of Agricultural Sciences in Anyang, Henan, China (longitude 36°06 N and latitude 114°21 E). Table 1 shows the climatic conditions during the cotton growing season from April to October in each year, which were obtained from the nearest meteorological station to the experimental site.

**Table 1:** The mean daily temperatures, cumulative hours of sunshine, and annual accumulated temperatures above 15°C from April 1^st^ to October 31^st^.

Year	Daily mean temperature (°C)	Sunshine (h)	Accumulated temperature above 15°C (°C)	Precipitation (mm)
2013	21.36	1318.36	4276.87	428.90
2014	22.03	1410.77	4484.50	450.50
2015	21.60	1425.92	4287.97	247.50

The study site was a medium loam soil that contained nitrogen, phosphorus, and potassium concentrations of 0.65, 0.01 and 0.15 g kg−1 soil, respectively. The experiment was designed randomly with cotton cultivar SCRC28 which was planted at six densities (15,000, 33,000, 51,000, 69,000, 87,000, and 105,000 plants ha^-1^) with 3 replicates. Each plot was 8.0 m wide and 8.0 m long and covered an area of 64.0 m^2^ with a 0.8 m row spacing. The sowing dates were April 17^th^, 2013, April 29^th^, 2014, and April 24^th^, 2015. Crop management of all the plots, including sowing, irrigation and fertilization, were the same. In addition, weeds, diseases, and pests were controlled in the cotton growing season to obtain the highest possible yields.

### Sampling and measurements

PAR interception (*IPAR*) was calculated by measuring the incident transmitted PAR (*TPAR*) and the reflected PAR (*RPAR*). *TPAR* and *RPAR* were measured using the spatial grid method at stable positions in six population-density plots with a portable 1.0-meter-line light quantum sensor (LA-191SA, LI-COR, Lincoln, NE, USA) and datalogger (LI-1400, LI-COR) every ten days after planting each year (Zhi et al. 2014). Then, the transmitted PAR rate (t*PAR*), reflected PAR rate (r*PAR*), and intercepted PAR rate (i*PAR*) were calculated using the following formulas:

$$tPAR=\frac{TPAR}{IPAR}$$

$$rPAR\frac{RPAR}{IPAR}$$

$$iPAR=\frac{IPAR-TPAR-RPAR}{IPAR}=1-tPAR-rPAR$$

The LI of the canopy was computed as follows, according to the Simpson 3/8 integration rules.

$$A_{\text{i}}=\frac{3\Delta x}{8}=(G_{i,1}+3G_{i,2}+3G_{i,3}+2G_{i,4}+\ldots +2G_{i,\;ncol-1}+G_{i,\;ncol})$$

$$Volume\approx \frac{3\Delta \text{y}}{8}(A_{1}+3A_{2}+3A_{3}+2A_{4}+\ldots +2A_{nocl-1}+A_{ncol})$$

where the coefficient vector is [5, 3, 3, 2, …, 3, 3, 2, 1], Δx is the vertical distance on the grid, Δy is the horizontal distance, G(i,j) is the grid node number, and volume is the total light volume of a certain cross-sectional area.

The LAI and dry matter mass were obtained on the same day that the PAR data were acquired. Two plants were randomly uprooted from each test plot, except from the two edge rows, and then they were divided into roots, stems, leaves and reproductive organs. A scanner (Phantom 9800xl, MiCROTEK, Shanghai, China) was used to take photos of the leaves, and leaf areas were determined using Image-Pro Plus 7.0 (Media Cybernetics, Rockville, MD, USA). The LAI was calculated as the total plant leaf area per unit area of land. The dry mass of roots, stems, leaves and reproductive organs was determined by drying at 80°C to a constant weight.

The k value and the optimum leaf area index (LAI) were calculated according to the following formula, which was reported by Xu [27].

$$\text{k}=\frac{-\text{ln}\frac{\text{t}PAR}{iPAR}}{\text{LAI}}$$

$${\text{LAI}}_{\text{OPT}}\text{=A}\times \frac{-1}{\text{K}}\times \text{ln}\;\text{E}$$

where A is a constant closely related to the solar radiation intensity. The E value is the ratio of the light intensity at which the light compensation point is reached relative to the average solar radiation intensity during the cotton growing season in a given area as described in detail previously [27].

### Statistical analysis

Correlation analysis was conducted to test the relationship between climatic factors and yield attributes. Means were compared using least significant difference tests at the p < 0.05 level of significance.

## Results

### Yield and yield components

There was no significant difference in seed cotton yield between the treatments with 105,000, 87,000 or 69,000 plants ha^-1^ in 2013 and 2015. However, in 2014, there was a significant difference between the treatments with 105,000 and 69,000 plants ha^-1^. This trend was also observed for lint yield, and the maximum values were obtained at the density of 105,000 plants ha^-1^ each year. The effects of planting density on boll weight and boll density were due to the indeterminate growth of cotton. As shown in Table 2, the boll weight decreased as the planting density increased. The opposite trend was observed for boll density. For example, as shown in Table 2, the maximum boll weight and minimum boll density appeared at a density of 15,000 plants ha^-1^. The lint percentage remained stable at approximately 43% in 2015. A significantly lower lint percentage was observed for a density of 105,000 plants ha^-1^ in 2013 and 2014.

**Table 2:** Yield and yield components of cotton under different planting densities from 2013 to 2015.

Planting year	Planting density treatment(plants ha^-1^)	Seed cotton yield (kg ha^-1^)	Lint cotton yield (kg ha^-1^)	Boll weight (g)	Boll density (ten thousand ha^-1^)	Lint percentage (%)
2013	15,000	3909.99d	1528.29c	6.04a	67.92e	38.99a
	33,000	4145.24c	1615.53b	5.89b	73.43de	38.87a
	51,000	4360.58ab	1674.22a	5.80b	85.11cd	38.69ab
	69,000	4320.96ab	1667.82a	5.84b	92.12bc	38.24b
	87,000	4379.66ab	1670.26a	5.72b	102.06ab	38.22b
	105,000	4454.41a	1696.57a	5.75b	109.72a	38.21b
2014	15,000	2932.82d	1278.96c	6.36a	45.78d	43.85ab
	33,000	4109.74c	1799.88b	5.93b	63.10c	44.01a
	51,000	4252.07c	1870.43b	5.84b	71.24b	43.96ab
	69,000	4529.24b	1989.49a	5.89b	88.07a	43.97ab
	87,000	4652.53ab	2068.67a	5.85b	87.19a	44.45a
	105,000	4815.53a	2090.63a	5.77b	90.32a	43.26b
2015	15,000	3724.21d	1614.76d	6.56a	63.01ab	43.39a
	33,000	4286.86c	1864.55c	6.43ab	77.95a	43.49a
	51,000	4533.74bc	1954.65bc	6.28abc	92.17ab	43.00a
	69,000	4644.00ab	1993.95abc	6.07bc	100.63bc	42.89a
	87,000	4785.12ab	2059.50ab	6.16c	112.95cd	42.98a
	105,000	4912.76a	2103.37a	6.11c	105.83d	42.78a

Means within a column followed by different letters are significantly different at p < 0.05

Comparing the same planting density treatments between years, seed cotton and lint cotton yields were higher in 2015 than in other years, except for the 87,000 plants ha^-1^ treatment in 2014. The maximum boll weights were 6.04 g in 2013, 6.36 g in 2014, and 6.56 g in 2015. The minimum boll weights were 5.72 g in 2013, 5.77 g in 2014, and 6.07 g in 2015. The boll density, which increased by 37.6% from 2014 to 2015 in the lowest-density treatment, was significantly different between 2014 and the other two years, as shown in Table 2. The effect of planting density on yield and yield components was significantly influenced by the differing climatic conditions of each year.

### Dry matter and harvest index

For a detailed analysis of the dry matter production (DM) for single plants and populations under different planting density treatments, five growth stages were studied, as shown in Table 3. There were no significant differences in DM, which was roughly equal to 1 g in all treatments, of single plants at the seedling stage except for the treatment of 105,000 plants ha^-1^ in 2013 and 2015. Because individual plant mass was so low, there was virtually no competition between plants at any planting density. However, the difference in DM between individual plants became more obvious in later stages, especially at the flower and boll-forming stage and boll-opening stage. Table 3 shows that the DM of single plants was highest in the lowest-density treatment: nearly three times higher for the 15,000 plants ha^-1^ treatment than for the 10500 plants ha^-1^ treatment at the boll-forming stage.

**Table 3:** Dry matter production (DM) and harvest index (HI) values of different stages of cotton at different planting densities from 2013 to 2015.

Planting year	Planting density treatment (plants ha^-1^)	Seedling stage DM		Bud stage DM		Preflower and boll-forming stage		Flower and boll-forming stage		Boll-opening stage		HI
		Single plant (g)	Population (kg)	Single plant (g)	Population (kg)	Single plant (g)	Population (kg)	Single plant (g)	Population (kg)	Single plant (g)	Population (kg)	
2013	15,000	1.34a	24.53d	20.47a	344.00d	111.16a	2036.46d	226.85a	4152.78c	344.99a	6339.35c	0.61a
	33,000	1.18ab	43.58cd	20.41a	763.56c	87.83b	2787.53c	154.33b	5765.00b	212.67b	7895.91bc	0.52b
	51,000	1.17ab	62.55bc	18.94a	1087.85bc	72.00c	3823.16b	143.50bc	7626.91a	181.45bc	9636.01ab	0.45c
	69,000	1.03ab	69.28ab	18.75a	1279.43b	66.46c	4492.68ab	116.01cd	7841.06a	149.53bc	10105.28ab	0.42c
	87,000	1.02ab	88.09a	16.96a	1322.85b	55.47d	4721.15a	95.00de	8082.45a	128.01c	10896.09a	0.40c
	105,000	0.92b	91.22a	15.55a	1687.72a	50.40d	5011.04a	84.79e	8433.21a	112.7c	11184.46a	0.39c
2014	15,000	0.96a	15.03e	19.60a	306.20e	131.37a	2052.59d	284.48a	4445.02d	341.31a	5332.97c	0.52a
	33,000	1.02a	39.54d	16.91b	653.73d	88.33b	3412.30c	193.64b	7486.69c	243.66b	9446.61b	0.45b
	51,000	1.06a	60.12c	15.77b	894.74 c	75.05bc	4266.38bc	162.03c	9213.33bc	201.00c	11417.96a	0.37b
	69,000	0.90a	65.52c	16.04b	1162.24b	65.91cd	4771.79ab	144.87d	10515.73ab	164.36d	11940.86a	0.37b
	87,000	0.93a	82.95b	15.71b	1402.22a	56.82cd	5048.32ab	121.67e	10841.86ab	136.27de	12151.33a	0.38b
	105,000	1.00a	103.15a	14.77b	1523.89a	53.7d	5544.31a	109.15f	11245.81a	123.04e	12685.98a	0.38b
2015	15,000	0.77a	14.09a	14.97a	273.89c	112.74a	2071.19d	265.43a	4852.08c	364.60a	6681.45b	0.55a
	33,000	0.65ab	24.40b	12.91a	480.45bc	89.78 ab	3358.55c	242.76a	9058.53b	230.32b	8587.42b	0.50a
	51,000	0.68ab	35.85c	11.40a	604.58bc	73.41bc	3895.01bc	202.98ab	10754.85ab	235.06b	12449.57a	0.38b
	69,000	0.62ab	41.55bc	12.87a	869.68ab	70.96bc	4800.13ab	157.62b	10652.40ab	195.55bc	13221.97a	0.35b
	87,000	0.55ab	46.91b	9.68a	826.10ab	63.14c	5359.77a	143.45b	12188.24ab	150.98c	12863.56a	0.38b
	105,000	0.65b	64.64a	11.99a	1191.82a	52.45c	5214.83ab	136.07b	13532.96a	147.89c	14716.45a	0.34b

Means within a column and within the same site followed by different letters are significantly different at p < 0.05

The population DM at the seedling stage increased significantly as planting density increased from 15,000 to 105,000 plants ha^-1^. However, there was no significant difference between the treatments of 51,000 and 69,000 plants ha^-1^. A similar trend was observed at the bud, preflower and boll-forming stages, but the difference became increasingly insignificant as the planting density increased. Differences in DM by population among all treatments between 51,000 and 105,000 plants ha^-1^ were insignificant during the boll-opening stage. Comparing the same planting density treatments across three years, the DM of single plants and populations at the seedling stage and bud stage were highest in 2013. However, the DM at later growth stages was highest in 2015.

The harvest index (HI) decreased significantly as planting density increased (Table 3). In 2014, the HI ranged from 0.52 to 0.38, with an average of 0.40 across all planting density treatments. In comparison, in 2015, the HI ranged from 0.55 to 0.34, with an average of 0.3.

### Planting density and LAI

**Fig 1:**
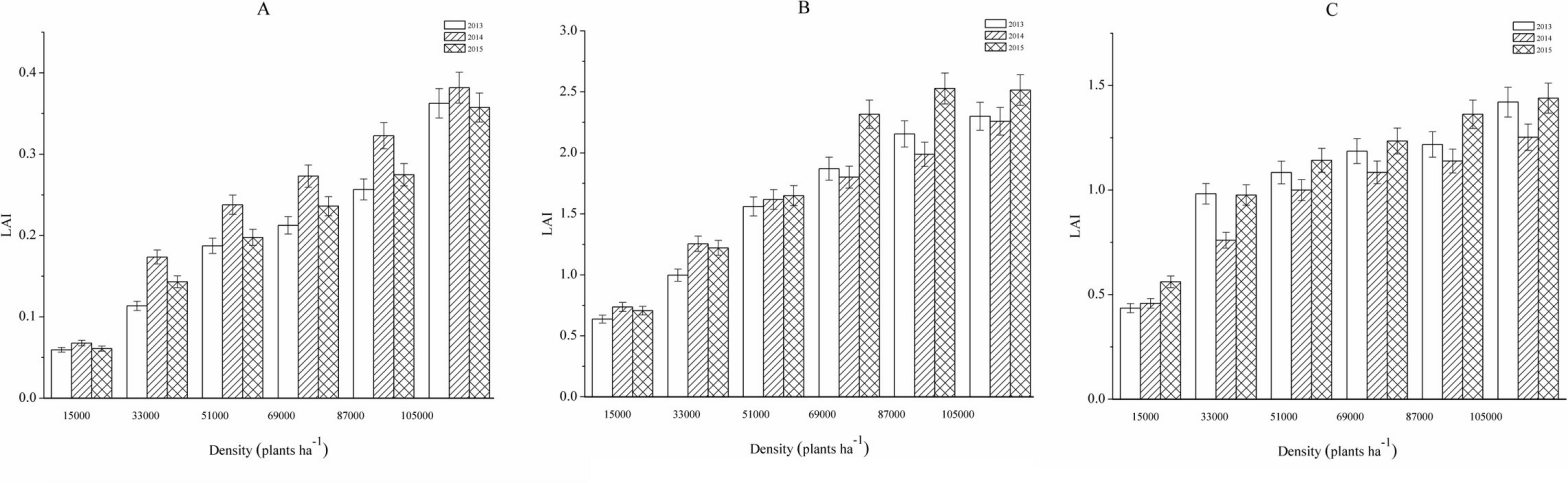
The leaf area indexes at the bud stage (A), flower and boll-forming stage (B) and boll-opening stage (C) under different planting densities from 2013 to 2015.

The LAI at the bud stage, flower and boll-forming stage and boll-opening stage were compared under different planting density treatments as Fig 1 shows. At the bud stage, the LAI, which was over five times higher for the 10500 plants ha^-1^ treatment than for the 15,000 plants ha^-1^ treatment in both 2014 and 2015, increased linearly as the planting density increased. The LAI increased with the growth of cotton and peaked at the flower and boll-forming stages. In the highest-density treatment, the LAI reached 2.39 in 2013, 2.25 in 2014, and 2.51 in 2015. In comparison, the LAI only reached 0.63 in 2013, 0.73 in 2014, and 0.70 in 2015 in the lowest-density treatment. With the advancement of the growth period, the LAI decreased significantly at the boll-opening stage as leaves fell at this point in the growing season. As shown in Fig 1, the LAI in the highest-density treatment decreased to 0.87 in 2013, 0.88 in 2014, and 0.7 in 2015. Comparison of LAI values for the same planting density treatments across years showed that the LAI was highest in 2014 at the bud stage. At the flower and boll-forming stage and boll-opening stage, the LAI was highest in 2015, except for the planting density treatments with 15,000 and 33,000 plants ha^-1^ at the flower and boll-forming stage.

As stated above, we defined the optimum LAI as the averaged LAI from 2013 to 2015 under the highest-density treatment with the highest cotton production. We calculated this as 2.39 at the flower and boll-forming stage. The average LAI values for the other treatments were 0.72, 1.24, 1.63, 2.06, and 2.26 from the lowest- to the highest-density treatments, respectively. The corresponding k values were calculated according to Formula (1). The average solar radiation intensity during the cotton growing season was 10×10^4^ lx in Henan Province. According to Formula (2) and the value of k, we calculated parameter A for the site. By following the steps above, we modified Formula (2) as follows: LAI opt = 0.24 × (−k /1 × lnE). The values of A were 0.12, 0.10, 0.13, 0.13, 0.14, and 0.19 from the lowest- to highest-density treatments, respectively. The results of the calculations indicated that parameter A was positively related to the planting density.

### Relationships between climatic conditions and the seed yield

Table 4 shows the climatic conditions of Henan Province from the sowing date to the boll-opening stage. Correlation analysis was conducted to determine the influence of climatic factors on seed cotton yield attributes (i.e., yield, yield components, DM, and LAI) at the optimum planting density (Table 5). The daily mean temperature was positively correlated with every yield attribute except boll density at the bud stage and flower and boll-forming stage, as shown in Table 5. The number of growing days was positively correlated with the seed cotton yield at the bud stage. However, during both the flower and boll-forming stage and boll-opening stage, the number of growing days was significantly negatively correlated with seed cotton yield, lint yield, DM, and boll weight. The diurnal temperature range was positively correlated with all yield attributes except for the boll density at all stages and with the LAI at the bud stage. The accumulated LI rate was significantly positively correlated with the yield attributes, especially DM. However, the accumulated LI rate was negatively correlated with boll density.

**Table 4:** Meteorological conditions during the cotton growing season from 2013 to 2015.

Year	Growth stage	Tm (°C)	Tr (°C)	DS	AL	Pr (mm)
2013	Bud stage	23.96	11.58	46	1.97	302
	Flower and boll-forming stage	26.23	8.38	77	10.83	34.8
	Boll-opening stage	25.87	10.91	130	24.78	28.4
2014	Bud stage	25.99	13.93	38	2.23	166
	Flower and boll-forming stage	26.59	10.61	65	11.08	46.1
	Boll-opening stage	25.15	11.88	116	25.99	160.3
2015	Bud stage	26.81	15.05	37	2.56	92.3
	Flower and boll-forming stage	27.14	12.90	70	13.17	29
	Boll-opening stage	24.26	13.02	120	27.45	35.4

Abbreviations: Tm, daily mean temperature; Ds, days after sowing; AL, accumulated LI rate. Pr, precipitation.

**Table 5:** Relationships between cotton yield attributes and meteorological conditions during bud stage, flower and boll-forming stage, and boll-opening stage.

Growth stage	Climatic variable	Seed cotton yield	Lint yield	Dry matter production	Boll weight	Boll density	LAI
Bud stage	Tm	0.9967*	0.9675*	0.9471*	0.7554*	-0.4178	0.0357
	Tr	0.9929*	0.9570*	0.9588*	0.7800*	-0.3825	-0.0027
	Ds	0.9949*	-0.9973*	-0.8736*	-0.6246	0.5751	0.11603
	AL	0.9209*	0.8389	0.9999*	0.9231	-0.1109	0.6558
Flower and boll-forming stage	Tm	0.9044*	0.8162*	0.9934*	0.9379*	-0.0706	0.6248
	Tr	0.9485*	0.8758*	0.9970*	0.8932*	-0.1819	0.7083*
	Ds	-0.8078*	-0.8982*	-0.5084*	-0.14486	0.9094*	-0.9847*
	AL	0.9878*	0.9446*	0.9686*	0.8025	-0.3483	0.8191
Boll-opening stage	Tm	0.9280*	-0.8489*	-0.9997*	-0.9158*	0.1294	-0.1101
	Tr	0.9333*	0.8564*	0.9992*	0.9099*	-0.1437	0.0958
	Ds	-0.8853*	-0.9528*	-0.6288	-0.2879	0.8389*	0.6854
	AL	0.9354	0.8525	0.9995*	0.9130	-0.1362	0.6748

Abbreviations: Tm, daily mean temperature; Ds, days after sowing; AL, accumulated LI rate.

*Correlation coefficients significant at p < 0.05.

## Discussion

### Yield, dry matter, and harvest index

Close planting is regarded as one key management technique to improve crop yield [34–36]. However, yield does not always increase as planting density increases, although other factors may be ideal [37]. Moreover, crop yields sometimes vary between areas with different climatic conditions, even with the same planting density and optimal management [27]. In this study, yields were greatest at a density of 105,000 plants ha^-1^, which was regarded as the optimum planting density for Anyang, Henan Province based on the range of the experimental data.

Cotton yield was affected by planting density in terms of the yield components, including boll weight and boll density. In this study, boll weight and boll density decreased with increasing planting density, which has also been noted in previous studies [16]. In addition, many studies have verified that high biomass is the foundation of high seed yield [38–40]. Therefore, improving DM accumulation during the growing season is necessary to increase seed yield [41]. In the present study, DM production increased gradually with the advancement of the growth period under all planting densities. Additionally, DM accumulation was positively correlated with cotton yield with a certain range of planting densities, as was previously observed by Dai [42]. In this study, DM increased as planting density increased, although the differences were not significant between higher-density treatments. The highest and lowest HI was observed at densities of 15,000 plants ha^-1^ and 105,000 plants ha^-1,^ respectively, supporting previous research that indicated that the HI decreased with increasing planting density [38]. However, there were small differences in the HI between years under the same planting density treatments. Therefore, we suggest that DM production played a more important role in achieving a high yield compared with the HI. These results are consistent with previous research performed on other crops [43–44].

### The optimum LAI and the LI

The LAI is an important factor that is closely related to LI, which influences the DM production of cotton [13]. Moreover, the LAI is the main physiological determinant of crop yield and can be used to reflect the crop production status to some degree. Therefore, maintaining the optimum LAI is the standard strategy for increasing light utilization efficiency and obtaining high seed cotton yield, especially at the flower and boll-forming stages [27]. In this study, the optimum LAI was calculated using a modified Monsi-formula. The optimum LAI was 2.36 at a density of 105,000 plants ha^-1^, while the highest cotton production and the optimal LAIs in the other treatments were 0.69, 1.16, 1.61, 1.99, and 2.22, respectively. In this study, high yield was accompanied by a high LAI, as was previously observed [45–46]. We obtained Formula (2), LAI opt = 0.24 × (−k /1 × lnE), for cotton in Henan Province at the flower and boll-forming stage. In addition, the values of parameter A were 0.12, 0.10, 0.13, 0.13, 0.14, and 0.19 from the lowest- to highest-planting density treatments, respectively. The results of the calculations indicated that parameter A was positively related to planting density.

### The relationship of climate to cotton yield, dry matter and the leaf area index

China has large and diverse cotton-producing areas with different climatic conditions. The optimum planting density changes significantly between different landscapes and different climates [47]. In this study, six different densities were established in Henan, which has more than 400 ha planted in cotton. The cotton yield, DM production, and LAI differed significantly under different planting densities. For example, the cotton seed yield in the lowest-density treatment was 25% lower than that in the 105,000 plants ha^-1^ treatment. Furthermore, the yield and yield components at the same planting density differed significantly between years. For example, cotton seed yields in the lowest-density treatments were 3909.99, 2932.82 and 3724.21 kg ha^-1^, respectively. These differences in yield can be attributed to the small differences in the climatic conditions between the three years.

The growth and development of cotton was significantly influenced by climatic conditions, including temperature, precipitation and solar radiation. Among the three factors, temperature has been proven to be the most important factor that influences crop growth [16]. In this study, the daily mean temperature was positively correlated with all of the yield attributes except for the boll density at the bud stage and flower and boll-forming stage. Moreover, LI was highly correlated with cotton production in a previous study [13]. Similarly, the accumulated LI rate was significantly positively correlated with all of the yield attributes at all growth stages, except for the boll density in our research, which indicates that the cotton yield will increase with increasing LI in the absence of other environmental stresses.

## Conclusion

The growth and development of cotton were influenced by climatic factors to a certain degree. In this study, the accumulated LI and diurnal temperature range were significantly positively. correlated with cotton production throughout the entire growing season. The number of growing days was positively correlated with seed cotton yield at the bud stage. However, during both the flower and boll-forming stage and boll-opening stage, the number of growing days was significantly negatively correlated with the yield and yield components. The optimum LAIs for Anyang, Henan were obtained at a planting density of 105,000 plants ha^-1^ and averaged 0.37 at the bud stage, 2.36 at the flower and boll-forming stage, and 1.37 at the boll-opening stage across three years. There were also differences in yield and yield components among the three years because of the differences in climatic conditions each year. This research provides guidance for managing cotton planting in Henan Province, China.
